# A New Tool to Aid the Differential Diagnosis of Physiological Remodelling from Cardiac Pathology When Assessing Left Ventricle, Left Atrial and Aortic Structure and Function in Male Arab and Black Paediatric Athletes

**DOI:** 10.3390/jcdd10020037

**Published:** 2023-01-20

**Authors:** Gavin McClean, Mathew G. Wilson, Nathan R. Riding, Guido Pieles, Victoria Watt, Carmen Adamuz, Anthony Shaw, Allan Harkness, Amanda Johnson, Keith P. George, David Oxborough

**Affiliations:** 1 Echocardiography Laboratory, St Bartholomew’s Hospital, Barts Health NHS, London EC1A 7BE, UK; 2 Echocardiography Laboratory, University College London Hospital, London NW1 2BU, UK; 3 Athlete Health and Performance Research Centre, Aspetar Orthopaedic and Sports Medicine Hospital, Doha 23833, Qatar; 4 Research Institute for Sport and Exercise Science, Liverpool John Moores University, Liverpool L3 3AF, UK; 5 Institute of Sport Exercise and Health (ISEH), University College London, London 1T 7HA, UK; 6 Bristol Medical School, University of Bristol, Bristol BS8 1UD, UK; 7 National Institute for Health Research (NIHR) Cardiovascular Biomedical Research Centre, Congenital Heart Unit, Bristol Royal Hospital for Children and Bristol Heart Institute, Bristol BS2 8ED, UK; 8 Department of Sports Medicine, Aspetar Orthopaedic and Sports Medicine Hospital, Doha 23833, Qatar; 9 Wythenshawe Hospital, Manchester University NHS Foundation Trust (MFT), Manchester M23 9LT, UK; 10 Colchester Hospital National Health Service Trust, Colchester CO4 5JL, UK; 11 Health Sciences Department, Manchester Metropolitan University, Manchester M15 6BH, UK

**Keywords:** paediatric, athlete’s heart, echocardiogram, ethnicity, sudden cardiac death

## Abstract

**Aim:** To determine if published Z-scores for left ventricular (LV), left atrial (LA) and aortic structure as well as indices of LV function (Doppler and TDI) in paediatric athletes and non-athletes are appropriate for application in male Arab and black paediatric athletes. If inappropriate, we aim to provide new nomograms and Z-scores for clinical application. **Methods:** 417 (297 Arab, 120 black) male paediatric (11–18 years) athletes, were evaluated by 2D echocardiography as per British Society of Echocardiography recommendations, and biological age (by radiological X-ray) assessment. Z-scores were tested by residual and correlation analysis together with visual inspection. New Z-scores involved allometric ($a*BSA^{\left(b+c*chronological\;age\right)}$) and second-order polynomial $\left(y=a*chronological\;age^{2}+b*chronological\;age+c\right)$ equations for measures of cardiac size and indices of LV function, respectively. **Results:** Residual linear regression, correlation analysis and visual inspection revealed published z-scores in white peri-pubertal footballers and paediatric non-athletes to be inappropriate for application in male Arab and black paediatric athletes. Residual linear regression revealed new Z-scores for measures of LV, LA and aortic root size to be independent of BSA, ethnicity, chronological and biological age. Residual linear regression revealed new Z-scores for measures of function to be independent of chronological age. **Conclusion:** Our new z-scores may aid differential diagnosis of suspected pathology versus physiology remodelling, in cardiac screening of the Arab and black paediatric athlete. Nomograms are provided to assist the tracking of the paediatric athlete necessitating annual follow-up and Excel z-score calculation to facilitate use in day-to-day practice.

## 1. Introduction

Increased left ventricular (LV) cavity, wall thickness, and mass are established manifestations of the paediatric athlete’s heart [1]. In some instances, however, this may overlap with phenotypic expression of dilated or hypertrophic cardiomyopathy or idiopathic LVH [2,3,4]; these cardiomyopathies collectively account for 62% of sudden cardiac deaths (SCDs) in paediatric footballers in the UK [5]. Echocardiography plays a vital role in the diagnostic differentiation between physiological and pathological remodelling, but necessitates population-specific reference values to reduce false-negative and -positive diagnoses, which may then serve to reduce the incidence of SCD in athletes. 

Until now, echocardiographic nomograms and Z-scores for LV and left atrial (LA) structure in paediatric athletes have been limited to white peri-pubertal footballers [6]. Previous studies, however, suggested that echocardiographic measurements in paediatric athletes are affected by ethnicity [4,7], chronological age [2,3], and biological (skeletal) age [8]. In extreme cases, biological age can vary by 6 years between two 9-year-old boys [9]. Indeed, significant racial variations exist in the chronological age of onset of puberty [10]. Finally, these Z-scores were derived from log-transformations, which may be limited, owing to an artificial distortion of the data [11]. 

Doppler and tissue Doppler imaging (TDI) have established roles in differential diagnosis between athlete’s heart and inherited cardiomyopathies [12,13]. Z-scores, however, are limited to paediatric non-athletes [14] and are likely not applicable to the paediatric athlete’s heart, which is typically enlarged with an augmented function [1]. Unfortunately, nomograms and Z-scores for black and Arab paediatric athletes—who present across academies throughout North America, Europe, and Asia—do not exist. Accordingly, it is time to answer calls for comprehensive nomograms, evaluating a complete dataset of 2D measurements in paediatric athletes [15]. 

We employed a rigorous statistical approach in order to evaluate the relationship that BSA, ethnicity, HR, chronological age, and biological age have to indices of LV, LA, and aortic structure, as well as LV function, in Arab and black paediatric athletes. We aim to determine whether current published nomograms and Z-scores from paediatric athletes [6] and non-athletes [14] are appropriate for application in male Arab and black paediatric athletes. If they are inappropriate, we aim to provide new nomograms and Z-scores for clinical application. 

## 2. Materials and Methods

### 2.1. Participants

**Figure 1:**
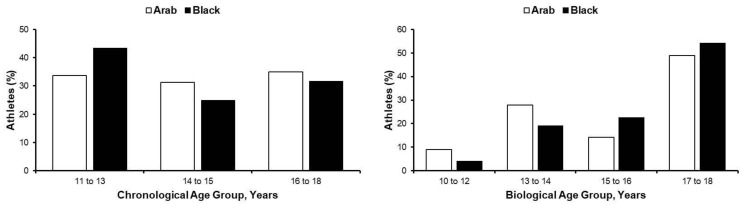
Participants chronological and biological age distribution.

Between 2013 and 2018, 297 Arab and 120 black male paediatric athletes who were registered with the Qatar Olympic Committee (training ≥6 h/week, aged 11–18 years) presented at Aspire Academy, Qatar for pre-participation screening. Demographic distribution of chronological and biological age categories are described in Figure 1; Table S1, with ≥120 participants per age group allowing for the determination of the 97.5th percentile for a given population [16], and thus the creation of new nomograms and Z-scores for clinical application. Participants’ ethical approval was provided by Anti-Doping Laboratory Qatar (IRB #E2013000003 and #E20140000012), with all parents/guardians providing informed consent, with self-declaration of ethnicity.

### 2.2. Preliminary Investigations 

#### Health Questionnaire and Electrocardiogram

Athletes completed a health questionnaire (with primary guardians) regarding family history and personal symptoms, together with anthropometric (height and body mass; body surface area (BSA) [17]) and blood pressure assessment. All athletes underwent a 12-lead ECG using a GE Mac 5500 (New York, NY, USA), with retrospective interpretation by GMC, applying international recommendations [18].

### 2.3. Echocardiography

All echocardiographic images were acquired by cardiologists and sonographers using IE33 (Philips, USA) and Artida (Cannon Medical Systems, Otawara, Japan) ultrasound systems with a 1.7–4 MHz phased array transducer, as per British Society of Echocardiography (BSE) recommendations [19]. Offline analysis was performed using commercially available software (QLab, Philips Medical, Amsterdam, The Netherlands; UltraExtend, Cannon Medical Systems, Otawara, Japan). 

#### D Echocardiography

LV internal diameter, septal and posterior wall thickness, aortic root diameter (at the sinus of Valsalva), and LA diameter were measured from 2D images in the parasternal long-axis view [19], allowing for LV mass calculation [19]. LV ejection fraction (LVEF) and LA volume were assessed as per Simpson’s biplane from the apical four-and two-chamber views. 

### 2.4. Doppler and Pulse-Wave Tissue Doppler Imaging

Transmitral Doppler assessment allowed measurement of peak early (E) and late diastolic (A) flow velocities, E deceleration time (DTec), and calculation of the E/A ratio. Pulse-wave TDI allowed measurement of peak myocardial velocities in early (e’) and late diastole (a’) as well as systole (s’) at the LV basal infero-septum and antero-lateral wall. Average E/e’ was calculated from both septal and lateral e’ [19].

### 2.5. Chronological and Biological Age Assessment

Chronological age was calculated as the difference between date of birth (as per original passport copy) and date of examination. Radiological hand-wrist imaging of the left-hand wrist using a Digital Diagnost (Philips, Andover, MA, USA) allowed biological age estimation as per the FELS method [20] by a single examiner, with previously demonstrated intra-class correlation coefficient of 0.998 [9]. 

### 2.6. Exclusion Criteria

Athletes with symptoms, or with physical examination, ECG and/or echocardiographic abnormalities suggestive of underlying cardiovascular pathology, underwent further evaluation as discussed previously [21]. Participants were excluded prior to enrolment in this study to provide a normative dataset. 

### 2.7. Statistical Analysis

Analysis was performed with SPSS software (V21.0; Chicago, IL, USA). A *p* value ≤ 0.05 demonstrated a significant effect; a *p* value ≤ 0.01 demonstrated a significant interaction among effects. Comparisons by ethnicity (black vs. Arab), employed a Student’s *t*-test for continuous variables and *x*^2^ test/Fisher’s exact tests for categorical variables (Supplementary File S1). 

To inform and support cardiac pre-participation screening, Z-scores of cardiac chambers, aortic root size, Doppler, and TDI were calculated from our cohort’s raw data using established equations from white peri-pubertal footballers [6] and paediatric non-athletes [14].

To provide new Z-scores of cardiac chamber and aortic root size to BSA and chronological age, an allometric equation $y=a*BSA^{\left(b+c*age\right)}$ was employed as guided by statistical analysis (Supplementary File S1; Tables S3 and S4) As per published Doppler and TDI velocity Z-score equations [14], second-order polynomial equations $\left(y=a*chronological\;age^{2}+b*chronological\;age+c\right)$ were employed. Correlation analysis determined the presence of a significant interaction (*p* ≤ 0.01) with BSA, chronological and biological age, ethnicity and HR. Appropriateness of fit was assessed by scatter plots of observations against BSA and chronological age respectively, with Z-score reference lines. If the interaction with ethnicity was significant, additional variance (R^2^) explained by the determination of ethnic-specific constants and coefficients were assessed, with an increase of <5% defined as clinically insignificant [22]. LVEF demonstrated no significant association with BSA, chronological age or biological age, with normal distribution, allowing for the determination of a lower reference limit for Z-scores [22]. 

Preliminary analysis revealed nonconstant variance of residual values across the entire range of BSA and chronological age, respectively, for most measures. Regressed SD (RSD) was calculated by linear regression of the scaled absolute value (multiplied by √(2/π)) [23]. Z-scores were then calculated, with measurements plotted against BSA and chronological age, respectively, with lines depicting the mean, +/−1, +/−2Z. 

## 3. Results

### 3.1. Demographics

The black athletes evaluated in this study descended from West Asia (42.5%), Eastern Africa (36%), Western Africa (25%) and Middle Africa (2.5%). The Arab athletes descended from West Asia (84.9%), North Africa (14.1%) East Africa (0.6%), and North America (0.3%). The athletes participated in 20 different sports, with football dominating (53.0%). The mean biological age (16.5 ± 1.8 vs. 16.0 ± 2.1 years, *p* ≤ 0.05) was greater in the black athletes, but mean chronological age (14.9 ± 1.9 vs. 15.0 ± 2.0 years) and BSA (1.6 ± 0.2 vs. 1.6 ± 0.3 m^2^) did not differ by ethnicity (Table S1). 

### 3.2. Left Ventricle, Left Atrial, and Aortic Root Size: Z-Scores from White Peri-Pubertal Footballers [6]

**Figure 2:**
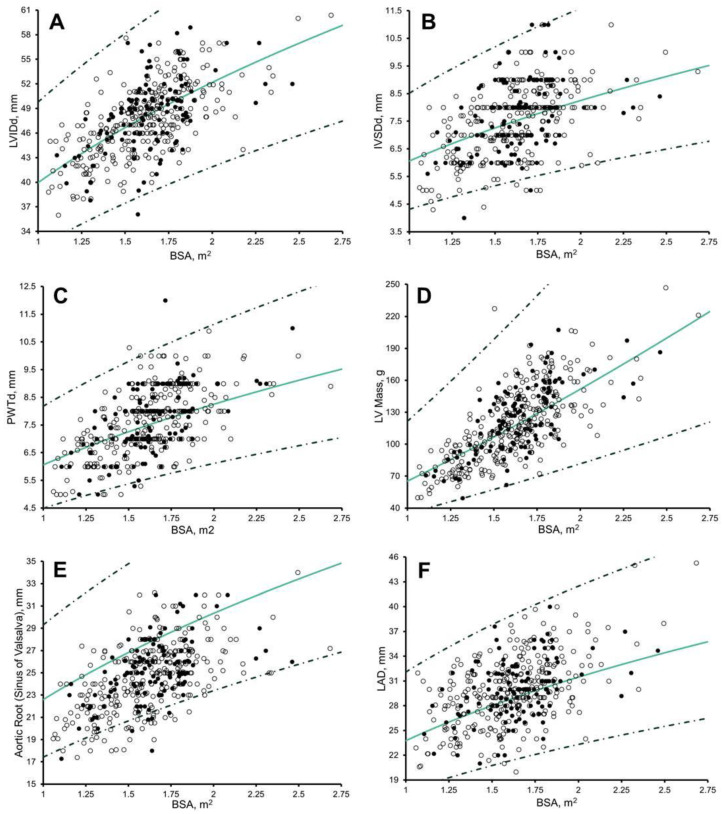
Scatter plots of: (**A**) Left Ventricle Internal Diameter (LVIDd); (**B**) Intraventricular Septal Wall thickness (IVSd), and; (**C**) Posterior Wall Thickness during end-diastole (PWTd); (**D**) LV Mass; (**E**) Atrial Root diameter, at the Sinus of Valsalva level during end-diastole; and (**F**) Left Atrial Dimension during end-systole to Body Surface Area (BSA) in 297 Arab (white dots) and 120 black (black dots). Solid green line, Z = 0; dashed dark green line, Z = 2 and −2, Cavarretta et al. [6] boundaries.

There remained significant correlation with BSA (r = 0.135 to 0.220, *p* ≤ 0.006) in 50%, biological age (r = 0.200 to 0.261, *p* ≤ 0.001) in 50%, and chronological age (r = 0.100 to 0.278, *p* ≤ 0.04) in 66.6% (Figure 2; Table S2) of measures after the application of Cavarretta et al. [6] Z-scores. 

### 3.3. Left Ventricle, Left Atrial, and Aortic Root Size: Arab and Black Paediatric Athlete Z-Scores

The allometric equation $y=a*BSA^{\left(b+c*chronological\;age\right)}$ revealed no residual association with BSA, ethnicity, chronological age or biological age across all measures of LV, LA, and aortic root size (Table S4).

**Figure 3:**
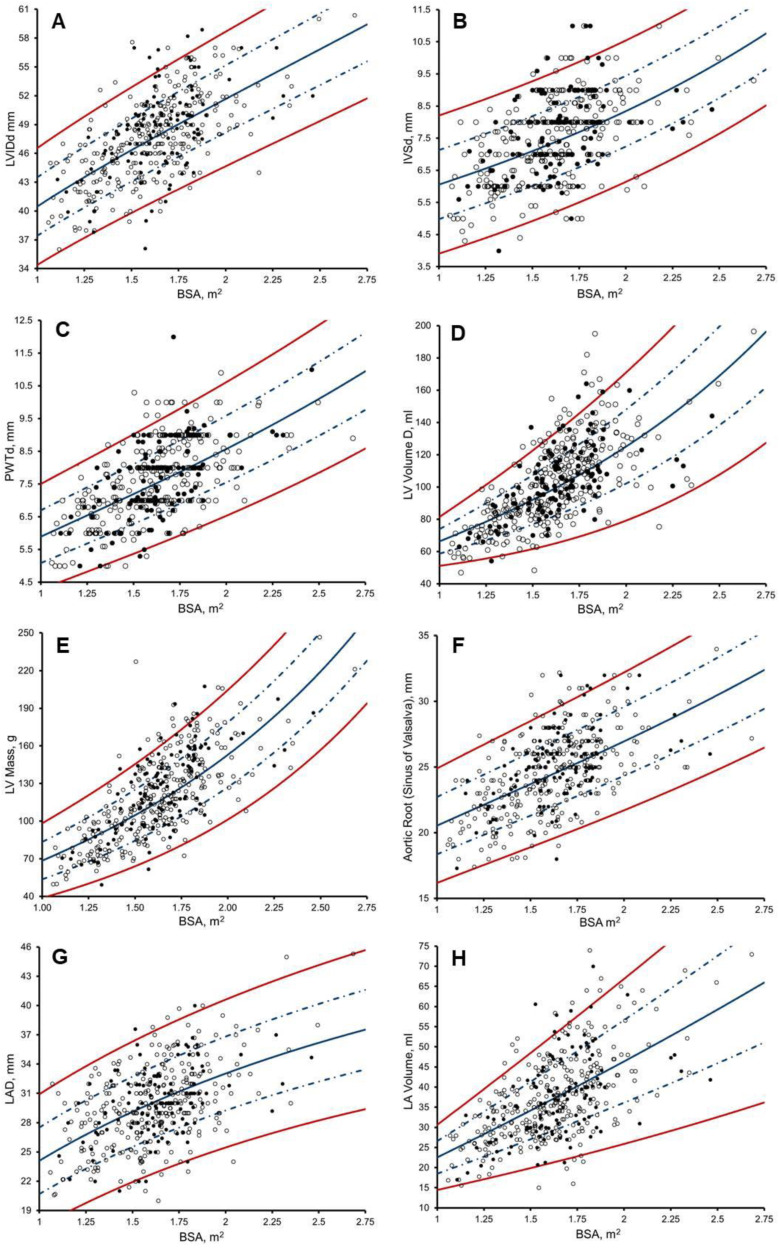
Scatter plots of: (**A**) Left Ventricle Internal Diameter (LVIDd); (**B**) Intraventricular Septal Wall thickness (IVSd); (**C**) Posterior Wall Thickness (PWTd), and; (**D**) LV Volume during end-diastole (LV VoI); (**E**) LV Mass; (**F**) Atrial Root diameter, at the sinus of Valsalva level during end-diastole; (**G**) Left Atrial Dimension (LAD) and; (**H**) LA Volume during end-systole to BSA in 297 Arab (white dots) and 120 black athletes (black dots), with predicted Z boundaries. Solid blue line, Z = 0; dashed blue line, Z = 1 and −1; solid red line, Z = 2 and −2.

LV, LA, and aortic root size are plotted against BSA with lines representing Z = 0, +/−1, and +/−2 (Figure 3), with RSD accounting for heteroscedasticity. Athlete Z-scores of LV, LA, and aortic root size can be calculated from Table S5 by using the specified *a*, *b*, *c* *d*, *e*, and *f* for that parameter of LV, LA, and aortic root size:

(1) $$z=\frac{obs-\left(a*BSA^{\left(b+c*chronological\;age\right)}\right)\;}{d+\left(e*BSA\right)+\left(f*chronological\;age\right)}$$

For a 13-year-old male paediatric athlete with a BSA of 1.76 m^2^ and a IVSd of 8.5 mm, the Z-score is 1.91, based on the values for *a* (6.055), *b* (−0.020), *c* (0.031), *d* (1.100), *e* (0.062), and *f* (−0.008). 

### 3.4. Pulsed-Wave Doppler and Tissue Doppler: Z-Scores from Paediatric Non-Athletes [14]

**Figure 4:**
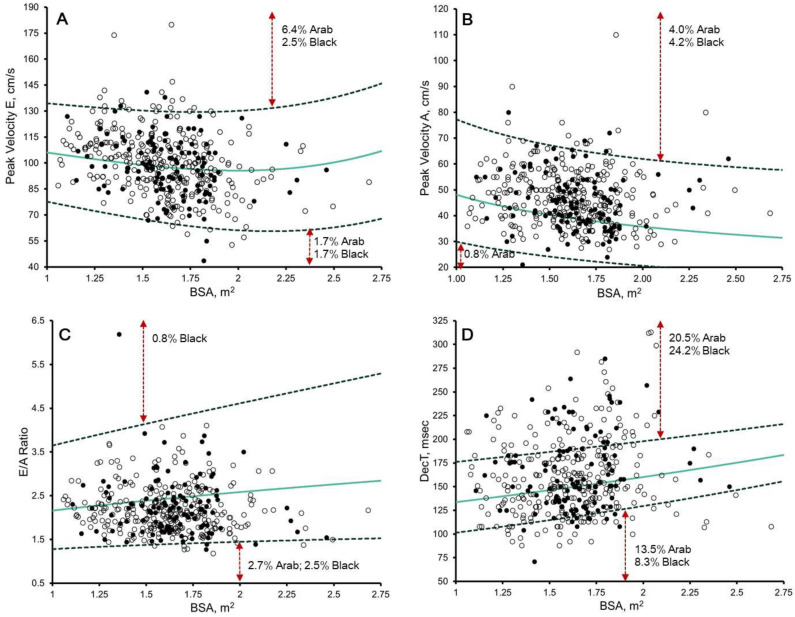
Scatter plots of: (**A**) peak E velocity; (**B**) peak A velocity; (**C**) E/A ratio; and (**D**) mitral E wave deceleration time (DecT) to body surface area (BSA) in 297 Arab (white dots) and 120 black (black dots). Solid green line, Z = 0; dashed dark green line, Z = 2 and −2, as per Dallaire et al. [14] proposed reference values. The percentages of Arab and black athletes exhibiting Z ≥ 2/≤ −2 are demonstrated.

**Figure 5:**
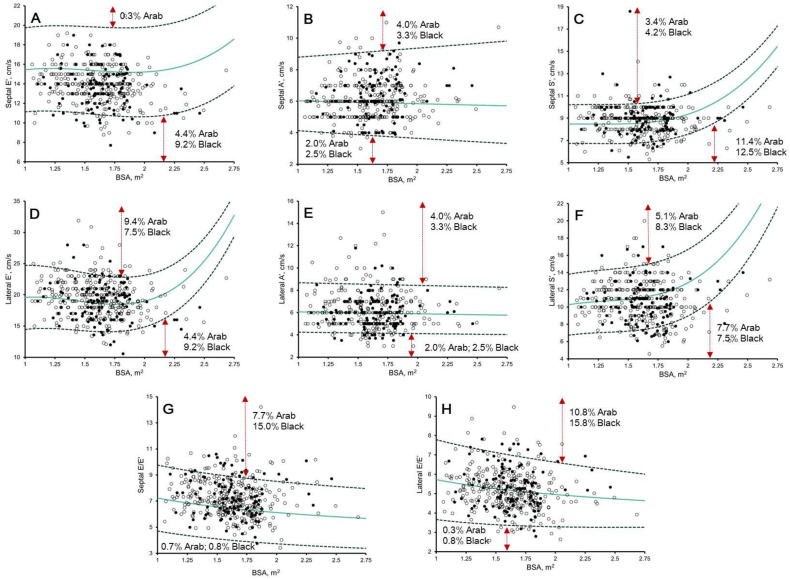
Scatter plots of: (**A**) septal myocardial velocity in early diastole (septal e’); (**B**) septal myocardial velocity in late diastole (septal a’); (**C**) septal myocardial velocity in systolic (septal s’); (**D**) lateral myocardial velocity in early diastole (lateral e’); (**E**) lateral myocardial velocity in late diastole (lateral a’); (**F**) lateral myocardial velocity in systolic (lateral s’); (**G**) septal E/e’ ratio; and (**H**) lateral E/e’ ratio to body surface area (BSA) in 297 Arab (white dots) and 120 black (black dots). Solid green line, Z = 0; dashed dark green line, Z = 2 and −2, as per Dallaire et al. [14] proposed reference values. The percentages of Arab and black athletes exhibiting Z ≥ 2/≤ −2 are demonstrated.

The upper and lower boundaries were often exceeded after the application of Dallaire et al. [14] Z-scores (Figure 4 and Figure 5). Significant correlation remained with chronological age (r = −0.116 to −0.285, *p* ≤ 0.01) in 58.3%, biological age (r = −0.190 to −0.239, *p* < 0.0001) in 33.3%, BSA (r = −0.421 to 0.140, *p* ≤ 0.004), in 50.0%, and HR (r = −0.162 to 0.233, *p* ≤ 0.01) in 58.3%. Black paediatric athletes presented significantly larger lateral e’ Z-scores than Arab paediatric athletes (mean difference = 0.315, *p* < 0.04) (Table S6).

### 3.5. LVEF, Doppler, and Tissue Doppler Imaging: Arab and Black Paediatric Athlete Z-Scores

Multivariable regression revealed a residual association between peak A velocity and biological age; septal TDI e’ and BSA; peak A velocity, E/A, and septal TDI a’ and HR; and lateral TDI e’ and ethnicity (Table S7). Determination of ethnic-specific *a* and *b* coefficients and *c* constants across all measures of Doppler and TDI velocity explained only an additional 2% of the variance (R^2^), which was considered clinically insignificant.

**Figure 6:**
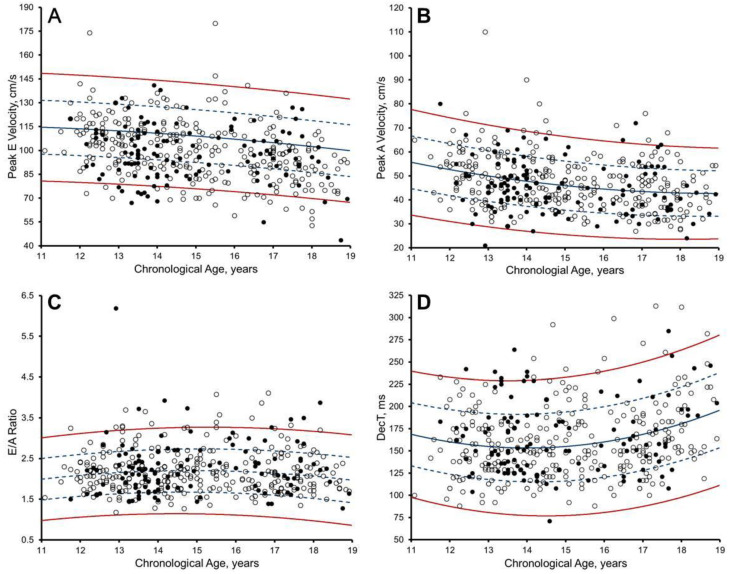
Scatter plots of: (**A**) peak E velocity; (**B**) peak A velocity; (**C**) E/A ratio; (**D**) mitral E wave deceleration time (DecT) to chronological age (years), in 297 Arab (white dots) and 120 black (black dots), with predicted Z boundaries. Solid blue line, Z = 0; dashed blue line, Z = 1 and −1; solid red line, Z = 2 and −2.

**Figure 7:**
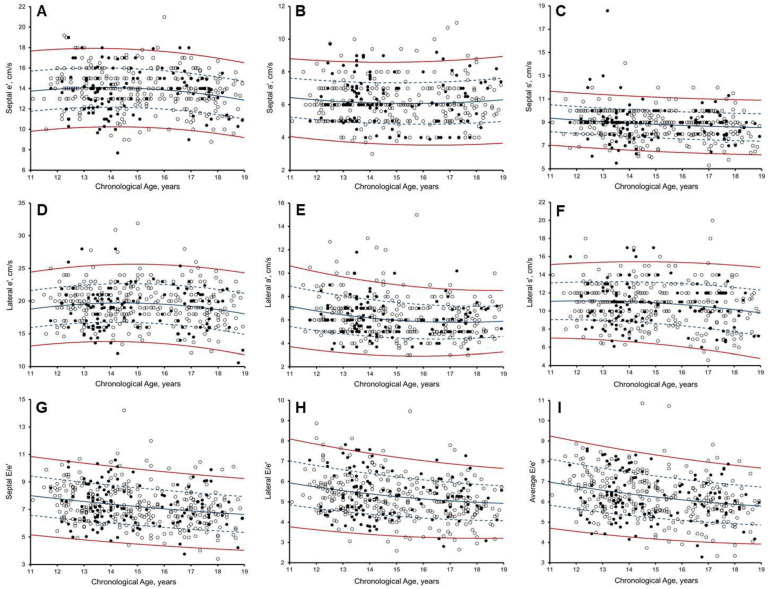
Scatter plots of: (**A**) Septal myocardial velocity in early diastole (Septal e’); (**B**) late diastole (Septal a’); (**C**) and systolic (Septal s’); (**D**) Lateral myocardial velocity in early diastole (LIral e’); (**E**) late diastole (Lateral a’); (**F**) and systolic (Lateral s’); (**G**) Septal E/e’ ratio; (**H**) Lateral E/e’ ratio; (**I**) Average E/e’ ratio to chronological age (years), in 297 Arab (white dots) and 120 black (black dots), with predicted Z boundaries. Solid blue line, Z = 0; dashed blue line, Z = 1 and −1; solid red line, Z = 2 and −2.

LVEF lower reference limits were determined to be 48.4%, allowing for determination of −2Z. Doppler and TDI velocities were plotted against chronological age with lines representing Z = 0, +/−1, and +/−2 (Figure 6 and Figure 7), with RSD accounting for heteroscedasticity. Athlete Z-scores can be calculated from Table S8 by using the specified *a*, *b*, *c*, *d*, and *e* for that parameter:

(2) $$z=\frac{obs-\left(\left(a*chronological\;age^{2}\right)+\left(b*chronological\;age\right)+c\right)}{d+\left(e*chronological\;age\right)}$$

For a 15-year-old male paediatric athlete with an average E/e’ of 4.3, the calculated Z-score is 1.87, based on the values for *a* (0.009), *b* (−0.418), *c* (10.485), *d* (1.413), and *e* (−0.025). 

## 4. Discussion

The correct differentiation of physiological cardiac enlargement or functional adaptation (owing to regular and sustained exercise) from cardiac pathology is paramount to the detection of athletes at risk of SCD during cardiac screening. We have demonstrated that current available nomograms and Z-scores from white paediatric athletes [6] and non-athletes [14] are inappropriate for application in male Arab and black paediatric athletes. For the first time, we present male-paediatric-athlete-specific nomograms and Z-scores for the assessment of LV, LA, and aortic size, together with Doppler, and TDI velocities, that are independent of BSA, ethnicity, chronological, and biological age. This removes the need for ethnic-specific normative ranges. In a clinical context, we propose a new tool for the differential diagnosis of physiological remodelling versus cardiac pathology in male Arab and black paediatric athletes. 

### 4.1. Left Ventricle Size in Male Arab and Black Paediatric Athletes

In a systematic review with meta-analysis [1], we observed that black paediatric athletes present disproportionately increased LV wall thickness (+12%) compared to white paediatric athletes, irrespective of chronological age. Indeed, Sheikh et al. [4] observed that almost 1 in 4 black paediatric athletes with LVH (>12 mm), were chronologically aged <16 years old. In view of test specificity, Sheikh et al. [4] proposed that established upper limits of LVH in black adult athletes (>15 mm), should be applied to black paediatric athletes [4]. We have previously demonstrated that ethnic-specific remodelling as a result of regular and sustained training extends to comparisons of black vs. Arab adult athletes [24]. Paediatric athletes, however, undergo significant anthropometric changes across chronological ages, with both acting as important determinants of cardiac size, as can be seen in their heteroscedastic relationship (Figure 3). We were thus unsurprised that log-transformed Z-scores [6] in male Arab and black paediatric athletes were not BSA-, biological-age- or chronological-age-independent in 50%, 50%, and 67%, respectively, across measures of LV size (Table S2). This finding is likely attributable to the artificial distortion of data [11] typically seen in log-transformed Z-scores. For example, a paediatric athlete, chronologically aged 11-years-old with a BSA of 1.5 m^2^ and an IVSd of 10 mm, would score 1.9 Z using Cavarretta et al. [6] Z-scores, and thus would not warrant further investigation or raise the suspicion of possible pathological remodelling. Conversely, this same athlete would score 2.8 Z using our new Z-scores, thus warranting further investigation and/or raise the suspicion of possible pathological remodelling. We found that allometric equations of LV size to BSA and chronological age, together with RSD to adjust for variable growth patterns, created Z-scores that were BSA-, biological-age- and chronological-age-independent, whilst removing the need for ethnic-specific normative ranges across all measures of LV size. 

### 4.2. Left Atrial Size in Male Arab and Black Paediatric Athletes

Until now, our understanding of ethnic-specific LA physiological remodelling in paediatric athletes has been limited to LAD assessment [4,7]. The LA, however, is a non-symmetrically shaped 3D structure; therefore, differentiation of physiological to pathological dilatation determined solely by LAD increases the risk of false-negative diagnosis [25,26]. Conversely, volumetric assessment accounts for LA dilatation in all planes, providing powerful prognostic value to a variety of inherited cardiomyopathies [25,26]. We therefore provide ethnic-, BSA-, chronological-age- and biological-age-independent Z-scores and nomograms of LAD and LA volume for clinical application in male Arab and black paediatric athletes. 

### 4.3. Aortic Arch in Male Arab and Black Paediatric Athletes

Athletes with suspected aortic disease are often discouraged from taking part in most competitive sports [27], owing to an increased risk of dissection/rupture and thus SCD. Yet 1 in 300 young athletes present with an enlarged aortic root diameter [27]. A disparity is evident between aortic root size in paediatric athletes and upper limits for non-athletes [6], necessitating paediatric-athlete-specific upper limits. Upper limits, however, are limited to white paediatric footballers. We found these log-transformed Z-scores [6] to be inappropriate upon visual inspection; they are likely to lack sensitivity. For example, a paediatric athlete, chronologically aged 12-years-old with a BSA of 1.25 m² and an aortic root of 30 mm, would score 1.5 Z using Cavarretta et al. [6] Z-scores, and thus would not warrant further investigation and/or raise the suspicion of pathological remodelling. Conversely, this same athlete would score 3.4 Z using our new Z-scores for application in male paediatric Arab and black athletes. 

### 4.4. Diastolic Function in Male Paediatric Arab and Black Athletes 

Unnithan et al. [28] demonstrated that early diastolic filling velocity (lateral e’) was not augmented at rest in Premier League academy footballers. We were therefore interested to see that 9.4% and 7.5% of Arab and black paediatric athletes, respectively, showed enhanced diastolic filling (Figure 5) using Z-scores from paediatric non-athletes [14]. Moreover, 2.0% of Arab and 2.5% of black paediatric athletes showed reduced late diastolic filling (lateral TDI a’), which correlated to HR, chronological age, and biological age. It is likely that regular and sustained training in paediatric athletes enhances early diastolic filling, allowing for the preservation of a high stroke volume during exercise, particularly at high heart rates. 

Although adult-athlete-specific cut-offs have been defined [12], chronological age is a strong determinant of myocardial relaxation, leading to calls for paediatric-athlete-specific thresholds [12]. We observed an inverse non-linear relationship between chronological age and TDI velocity (Figure 6 and Figure 7). Until now, LV function in paediatric athletes has been understood to be independent of ethnicity [4,7]. Investigations, however, were limited to the assessment of Doppler velocity, precluding an understanding of regional relaxation and contractile function. Despite adjustment for chronological age, new Z-scores for lateral TDI e’ velocity remained dependent on ethnicity, albeit at a power considered clinically insignificant, such that ethnic-specific Z-scores are not necessitated. 

### 4.5. Systolic Function in Male Paediatric Arab and Black Athletes

Secondary to enhanced diastolic function, paediatric athletes typically present with a ‘reduced’ LV ejection fraction (LVEF); observed to be as low as 50% [7]. Thus creates an illusion of an abnormal pump. A manifestation attributable to increased LV end-diastolic volumes from regular and sustained training, enabling the delivery of the same stroke volume at rest with reduced work. Accordingly, we present new paediatric-athlete-specific LVEF Z-scores to account for this sluggish pump patten. LVEF, however, assesses only radial function, with longitudinal functional impairment often preceding radial systolic dysfunction, allowing for differentiation between athlete’s heart and inherited cardiomyopathies [29,30]. Limitations highlighted by investigations in pathological hypertensive patients diagnosed with heart failure, but with preserved EF [31]. 

Conversely, longitudinal systolic function assessed at the mitral annular tissue level (TDI s’) is regarded to be a less load-dependent measure of function. We were therefore surprised that non-athlete Z-scores [14] indicated reduced systolic function in 7.7% of Arab and 7.5% of black paediatric athletes, respectively (Figure 5). We therefore provide athlete-specific reference values for the assessment of s’ within Arab and black paediatric athletes, which may allow for increased specificity in the detection of reduced longitudinal function [12,13].

### 4.6. When to Consider HR in Assessing LV Function in Male Paediatric Arab and Black Athletes 

As previously observed, [14] a significant residual association existed between HR and Z-scores of peak A velocity, E/A, and septal TDI a’. We, however, chose not to normalise to HR, owing to the complex relationship of HR and blood flow to tissue dynamics. Although our nomograms and Z-scores may be influenced by HR, we believe that they still offer the benefit of being adjusted for chronological age. With this in mind, nomograms and Z-scores with HR dependency should be interpreted with caution, especially in paediatric athletes with an unusually low or high resting HR for their respective chronological age and/or training status. For example, the residual slope of the E/A Z-score versus HR was −0.009, meaning that for every increase of 10 beats.min^−1^, there was a reduction in E/A by 0.01.

### 4.7. Clinical Implications: How to Apply in the Clinic Today

Differential diagnosis between physiological remodelling and an inherited cardiomyopathy that may predispose male Arab and black paediatric athletes to SCD is challenging. We have provided Z-scores for the assessment of LV, LA, and aortic size and function that may aid such differentiation. Timely manual calculation of Z-scores for all measures included within the minimum dataset of a TTE examination [19] is likely impractical. All Z-scores generated through this study are publicly available in our excel z-score calculator (Excel S1). In application, the attending clinician/echocardiographer may enters the patients’ age, height, weight, and the respective measured value to receive an automated Z-score. This is a useful tool when tracking allometric growth in the paediatric athlete necessitating annual follow-up. 

### 4.8. Limitations

Our population were exclusively healthy Arab and black male paediatric athletes, so the application to female and white paediatric athletes is limited. There was no consideration of the impact of geographical origin [32], as the relative impact of ethnicity was considered clinically insignificant after application of our Z-score equations. Whilst we recruited only athletes who were registered with the Qatar Olympic Committee and training ≥6 h/week, we did not define fitness (such as aerobic capacity). Finally, measures of speckle-tracking echocardiography were not included, as measures were limited to standard echocardiographic assessment.

## 5. Conclusions

For the first time, we present male paediatric athlete specific nomograms and Z-scores for the assessment of LV, LA, and aortic size, Doppler, and TDI velocities, which are BSA-, ethnicity-, chronological-age-, and biological-age-independent, removing the need for ethnic-specific normative ranges. These data may prove useful for differential diagnosis in cardiac screening of paediatric athletes. Nomograms are provided to assist in the tracking of paediatric athletes where annual follow-up and online calculation are necessitated, to facilitate use in day-to-day practice.

## Data Availability

Data will be made available upon receipt of written request.

## Supplementary Materials

The following supporting information can be downloaded at: https://www.mdpi.com/article/10.3390/jcdd10020037/s1. **Table S1.** Anthropometric data of Arab and black paediatric athletes by chronological age group; **Table S2.** Correlation analysis for Caverreta et al. [6] Z-scores of left ventricle, atrial, and aortic root size in the male Arab and black paediatric athlete; **Table S3.** Residual association for measures of left ventricle, atrial, and aortic root Size in the male Arab and black paediatric athlete $\left(y=a*BSA^{b}\right)$; **Table S4.** Residual association for measures of left ventricle, atrial, and aortic root size in the male Arab and black paediatric athlete $\left(y=a*BSA^{\left(b+c*chronological\;age\right)}\right)$; **Table S5.** Equation, predicted mean, and regressed standard deviation for measurements of left ventricle, atrial, and aortic root size in the male Arab and black paediatric athlete; **Table S6.** Correlation analysis for Dallaire et al. [14]. Z-score of Doppler and tissue Doppler imaging velocities in the Arab and black male paediatric athlete; **Table S7.** Residual association for left ventricle Doppler and tissue Doppler imaging velocities in the male Arab and black paediatric athlete $\left(y=a*chronological\;age^{2}+b*chronological\;age+c\right)$; **Table S8.** Equations, predicted mean and regressed standard deviation parameters of left ventricle Doppler and tissue Doppler imaging velocities in the Arab and black paediatric athlete. **Supplementary File S1:** Statistical Analysis Supplement. **Excel S1:** Z-score calculator when assessing left ventricle, left atrial, and aortic structure and function in male Arab and black paediatric athletes. Reference [33] is cited in the supplementary materials.
