# Supplementary material for: A New Tool to Aid the Differential Diagnosis of Physiological Remodelling from Cardiac Pathology When Assessing Left Ventricle, Left Atrial and Aortic Structure and Function in Male Arab and Black Paediatric Athletes
Source: J Cardiovasc Dev Dis. 2023 Jan 20;10(2):37. doi: 10.3390/jcdd10020037 (PMC9963999; doi:10.3390/jcdd10020037)
Supplement: Supplementary file 1 [file jcdd-10-00037-s001.zip › Data Supplement - Tables.pdf]

Data Supplements

**Supplement Table S1.** Anthropometric data of Arab and black paediatric athletes by chronological age group.								
Chronological Age Group, years	Group	*N*	%	Biological Age, years)	BSA, m^2	Systolic BP, mmHg	Diastolic BP, mmHg	Heart Rate, beats.min^{-1}
11–13	Total	*152*	33.8	14.3 ± 1.7	1.44 ± 0.21	114 ± 10	64 ± 9	72 ± 11
	Arab	*100*		13.8 ± 1.5	1.40 ± 0.20	112 ± 9	63 ± 8	72 ± 11
	Black	*52*		15.3 ± 1.7**	1.52 ± 0.21**	118 ± 10**	66 ± 10	70 ± 12
14–15	Total	*126*	36.2	16.3 ± 1.4	1.64 ± 0.20	117 ± 9	65 ± 9	66 ± 11
	Arab	*93*		16.2 ± 1.4	1.63 ± 0.20	116 ± 9	64 ± 8	66 ± 11
	Black	*30*		16.7 ± 1.5	1.66 ± 0.20	119 ± 9	67 ± 9	66 ± 8
16–18	Total	*142*	30.0	17.8 ± 0.5	1.80 ± 0.18	119 ± 10	65 ± 9	61 ± 10
	Arab	*104*		17.8 ± 0.5	1.80 ± 0.19	119 ± 10	66 ± 10	62 ± 10
	Black	*38*		17.9 ± 0.3	1.79 ± 0.17	120 ± 9	65 ± 9	60 ± 10

Values are mean ± standard deviation; BSA, body surface area; BP, blood pressure; beats.min^{-1}, beats per minute; cm, centimetres; kg, kilograms; m^2, $meters^2$; mmHg; millimetre of mercury.

* $p \leq 0.01$, significantly more prevalent or greater in black than Arab athletes.

** $p \leq 0.001$, significantly more prevalent or greater in black than Arab athletes.

Supplement Table S2. Correlation analysis for Caverreta et al. [1]. Z-scores of left ventricle, atrial, and aortic root size in paediatric male Arab and black athletes.

	Correlation Analysis							
	BSA		Chronological Age		Biological Age		Race	
Parameter	R	P Value	R	P Value	R	P Value	Mean Diff	P Value
LVIDd, mm	−0.031	0.521	−0.019	0.702	−0.032	0.513	−0.072	0.308
IVSd, mm	0.135	0.006	0.231	<0.0001	0.214	<0.0001	−0.014	0.881
PWTd, mm	0.220	<0.0001	0.278	<0.0001	0.261	<0.0001	−0.151	0.115
LV mass, g	0.146	0.003	0.227	<0.0001	0.200	<0.0001	−0.099	0.136
LAD, mm	0.036	0.463	0.100	0.041	0.141	0.004	−0.112	0.179
Aortic Root, mm	0.034	0.492	−0.001	0.991	−0.029	0.549	−0.017	0.846

Diff, difference; g, grams; LVIDd, left ventricle Internal diameter; IVSd, intraventricular septal wall thickness; PWTd, posterior wall thickness during diastole; LAD, left atrial diameter during end-systole; ml, millilitres; mm, millimetres.

Supplement Table S3. Residual association for measures of left ventricle, atrial, and aortic root size in paediatric male Arab and black athletes ($y = a * BSA^b$).

	Residual Association							
	BSA		Chronological Age		Biological Age		Race	
Parameter	Slope	P Value	Slope	P Value	Slope	P Value	Mean Diff	P Value
LVIDd, mm	−0.020	0.977	0.243	0.072	−0.132	0.316	−0.378	0.309
IVSd, mm	0.008	0.971	0.110	0.010	−0.016	0.704	−0.165	0.166
PWTd, mm	0.012	0.951	0.100	0.007	−0.025	0.480	0.004	0.968
LV Vol D, ml	1.866	0.613	2.234	0.002	−0.249	0.724	0.126	0.950
LVM, g	2.771	0.546	3.395	0.000	−0.864	0.323	−3.211	0.203
Aortic Root, mm	−0.019	0.970	0.082	0.399	0.113	0.232	−0.298	0.266
LAD, mm	0.020	0.977	0.035	0.801	−0.158	0.238	−0.025	0.948
LA Vol, ml	0.149	0.929	0.145	0.666	−0.116	0.725	0.685	0.458

Diff, difference; g, grams; LVIDd, left ventricle internal diameter; IVSd, Intraventricular septal wall thickness; PWTd, posterior wall thickness; LV Vol D, LV volume during end-diastole; LAD, left atrial diameter; LA Vol, LA volume during end-systole; ml, millilitres, mm, millimetres.

Supplement Table S4. Residual association for measures of left ventricle, atrial, and aortic root size in paediatric male Arab and black athletes ($y = a * BSA^{(b+c*chronological\ age)}$).

	Residual Association							
	BSA		Chronological Age		Biological Age		Race	
Parameter	Slope	P Value	Slope	P Value	Slope	P Value	Mean Diff	P Value
LVIDd, mm	0.036	0.739	−0.202	0.813	0.007	0.931	−0.461	0.212
IVSd, mm	0.035	0.299	−0.167	0.534	0.040	0.135	−0.217	0.063
PWTd, mm	0.043	0.148	−0.191	0.412	0.031	0.174	−0.033	0.747
LV Vol D, ml	0.527	0.361	−0.924	0.839	0.838	0.060	−0.949	0.630
LVM, g	0.816	0.255	−1.471	0.794	0.936	0.091	−4.565	0.062
Aortic Root, mm	0.063	0.420	−0.331	0.588	0.125	0.037	−0.393	0.139
LAD, mm	−0.051	0.851	0.381	0.858	−0.035	0.868	0.634	0.492
LA Vol, ml	−0.065	0.558	0.332	0.702	−0.113	0.184	0.012	0.975

Diff, difference; g, grams; LVIDd, left ventricle internal diameter; IVSd, intraventricular septal wall thickness; PWTd, posterior wall thickness; LV Vol D, LV volume during end-diastole; LAD, left atrial diameter; LA Vol, LA volume during end-systole; ml, millilitres; mm, millimetres.

Supplement Table S5. Equation, predicted mean, and regressed standard deviation for measurements of left ventricle, atrial, and aortic root size in paediatric male Arab and black athletes.

		Predicted Mean Parameters			Regressed SD Parameters		
Parameter	Equation	*a*	*b*	*c*	*D*	*E*	*f*
LVIDd, mm	Allometric	40.469	0.228	0.008	2.276	0.250	0.047
IVSd, mm	Allometric	6.055	−0.020	0.031	1.100	0.062	−0.008
PWTd, mm	Allometric	5.891	0.214	0.021	0.562	0.194	0.005
LV Vol D, mm	Allometric	66.236	0.20	0.046	−10.763	13.239	0.465
LVM, g	Allometric	68.499	0.396	0.049	1.230	8.960	0.429
Aortic Root, mm	Allometric	20.531	0.128	0.017	1.719	0.431	0.003
LAD, mm	Allometric	24.094	0.533	−0.005	3.950	1.035	−0.143
LA Vol, ml	Allometric	22.538	0.948	0.006	−4.254	4.719	0.326

G, grams; LVIDd, left ventricle internal diameter during diastole; IVSd, intraventricular septal wall thickness during diastole; PWTd, posterior wall thickness during diastole; LV Vol D, LV volume during diastole; LAD, left atrial diameter; LA Vol, left atrial volume during systole; ml, millilitres; mm, millimetres; SD, standard deviation.

Supplement Table S6. Correlation analysis of Dallaire et al. [2]. Z-score of Doppler and tissue Doppler imaging velocities in paediatric male Arab and black Athletes.

	Correlation Analysis									
	Chronological Age		Biological Age		BSA		Heart Rate		Race	
Parameter	R	P Value	R	P Value	R	P Value	R	P Value	Mean Diff	P Value
Peak E Velocity, cm/s	−0.267	<0.0001	−0.239	<0.0001	−0.221	<0.0001	0.128	0.009	0.136	0.251
Peak A Velocity, cm/s	−0.116	0.017	−0.001	0.985	0.097	0.047	0.233	<0.0001	0.167	0.077
E/A ratio	−0.100	0.040	−0.190	<0.0001	−0.260	<0.0001	−0.122	0.013	−0.071	0.454
DecT, ms	0.096	0.051	0.018	0.716	−0.048	0.329	−0.162	0.001	−0.219	0.333
Septal e’, cm/s	−0.077	0.116	−0.082	0.094	−0.154	0.002	0.072	0.142	0.110	0.255

Commented [GT1]: Please check that the intended meaning has been retained.

Commented [MG(HNT2R1]: This is fine.

Septal a’, cm/s	−0.001	0.989	0.087	0.075	0.140	0.004	0.105	0.032	−0.133	0.205
Septal s’, cm/s	−0.251	<0.0001	−0.239	<0.0001	−0.336	<0.0001	0.150	0.002	−0.090	0.603
Septal E/e’	−0.116	0.018	−0.058	0.235	0.037	0.456	0.011	0.823	−0.020	0.872
Lateral e’, cm/s	−0.040	0.420	−0.087	0.077	−0.211	<0.0001	−0.088	0.071	0.315	0.039
Lateral a’, cm/s	−0.144	0.003	−0.111	0.023	−0.033	0.501	0.140	0.004	0.107	0.325
Lateral s’, cm/s	−0.285	<0.0001	−0.222	<0.0001	−0.421	<0.0001	0.113	0.022	0.157	0.300
Lateral E/e’	−0.147	0.003	−0.058	0.240	−0.003	0.947	0.125	0.011	−0.145	0.237

Cm/s, centimetres/second; DecT, deceleration time; Diff, difference; msec, milliseconds.

Supplement Table S7. Residual association for left ventricle Doppler and tissue Doppler imaging velocities in paediatric male Arab and black athletes ($y = a * chronological\ age^2 + b * chronological\ age + c$).

	Residual Association									
	Chronological Age		Biological Age		BSA		HR		Race	
Parameter	Slope	P Value	Slope	P Value	Slope	P Value	Slope	P Value	Mean Diff	P Value
Peak E Velocity, cm/s	0.000	1.000	0.227	0.731	−9.688	0.055	−0.003	0.968	3.059	0.098
Peak A Velocity, cm/s	0.000	1.000	−1.017	0.000	−1.328	0.012	0.045	0.000	2.312	0.043
E/A ratio	0.000	1.000	−0.024	0.265	−0.174	0.292	−0.009	0.001	−0.073	0.237
DecT, msec	0.000	1.000	−1.689	0.268	13.438	0.249	−0.450	0.014	−6.107	0.153
Septal e’, cm/s	0.000	1.000	0.109	0.153	−1.601	0.006	0.009	0.311	0.304	0.155
Septal a’, cm/s	0.000	1.000	0.033	0.501	0.657	0.084	0.017	0.004	−0.221	0.117
Septal s’, cm/s	0.000	1.000	−0.004	0.938	0.304	0.424	0.012	0.044	−0.064	0.649
Septal E/e’	0.000	1.000	−0.051	0.339	0.236	0.567	−0.008	0.190	0.029	0.849

Lateral e’, cm/s	0.000	1.000	−0.129	0.267	−0.922	0.298	−0.029	0.036	0.847	0.009
Lateral a’, cm/s	0.000	1.000	0.014	0.819	0.348	0.468	0.013	0.094	0.183	0.298
Lateral s’, cm/s	0.000	1.000	0.196	0.029	−0.986	0.149	0.019	0.075	0.308	0.219
Lateral E/e’	0.000	1.000	0.046	0.240	−0.274	0.358	0.007	0.156	−0.091	0.406
Average E/e’	0.000	1.000	−0.003	0.946	−0.019	0.951	−0.001	0.854	−0.031	0.787

Cm/s, centimetres/second DecT, deceleration time; Diff, difference; msec, milliseconds.

Supplement Table S8. Equations, predicted mean, and regressed standard deviation parameters of left ventricle Doppler and tissue Doppler imaging velocities in paediatric male Arab and black athletes.

		Predicted Mean Parameters			Regressed SD Parameters	
Parameter	Equation	*a*	*b*	*c*	*D*	*e*
Peak E Velocity, cm/s	Polynomial (Second Order)	−0.155	1.597	110.963	17.903	−0.087
Peak A Velocity, cm/s	Polynomial (Second Order)	0.202	−7.686	115.752	13.125	−0.193
E/A ratio	Polynomial (Second Order)	−0.014	0.417	−0.901	0.442	0.006
DecT, msec	Polynomial (Second Order)	1.710	−47.889	488.577	26.138	0.850
Septal e’, cm/s	Polynomial (Second Order)	−0.045	1.239	5.547	2.153	−0.017
Septal a’, cm/s	Polynomial (Second Order)	0.018	−0.557	10.382	1.000	0.017
Septal s’, cm/s	Polynomial	0.008	−0.340	12.126	1.136	0.002

	(Second Order)					
Septal E/e’, cm/s	Polynomial (Second Order)	0.007	−0.381	11.352	1.589	−0.015
Lateral e’, cm/s	Polynomial (Second Order)	−0.080	2.308	3.057	2.412	0.038
Lateral a’, cm/s	Polynomial (Second Order)	0.035	−1.210	16.250	2.299	−0.052
Lateral s’, cm/s	Polynomial (Second Order)	−0.029	0.710	6.767	1.351	0.061
Lateral E/e’, cm/s	Polynomial (Second Order)	0.011	−0.456	9.618	1.391	−0.028
Average E/e’, cm/s	Polynomial (Second Order)	0.009	−0.418	10.485	1.413	−0.025

Cm/s, centimetres/second; DecT, deceleration time; msec, milliseconds; SD, standard deviation.
