# Supplementary material for: A New Tool to Aid the Differential Diagnosis of Physiological Remodelling from Cardiac Pathology When Assessing Left Ventricle, Left Atrial and Aortic Structure and Function in Male Arab and Black Paediatric Athletes
Source: J Cardiovasc Dev Dis. 2023 Jan 20;10(2):37. doi: 10.3390/jcdd10020037 (PMC9963999; doi:10.3390/jcdd10020037)
Supplement: Supplementary file 1 [file jcdd-10-00037-s001.zip › Supplementary File S1-Statistical analysis supplement.pdf]

***Statistical Analysis* Supplement 1**

Statistical Analysis

Analysis was performed with SPSS software (version 21.0; Chicago, IL, USA) to provide Z-scores of LV size, LA size, aortic root size, Doppler, and TDI velocities. A P value ≤0.05 determined a significant effect, and a P value ≤0.01 determined a significant interaction among effects. Comparisons by race (black vs. Arab) employed a Student's t-test for continuous variables, and x^2 test/Fisher's exact tests were used for categorical variables.

To inform and support clinical pre-participation screening, we calculated the Z-scores of cardiac chamber sizes, aortic root size, Doppler, and TDI from our cohort's raw data using established Z-score equations from white peri-pubertal football players [1] and paediatric non-athletes [2]. Correlation analysis determined the presence of a significant interaction (p≤0.01) to BSA, chronological and biological age, race, and HR. Scatter plots of observations against BSA and chronological age, respectively, with Z-score reference values, assessed the appropriateness of fit.

To provide normalised measures of cardiac chamber and aortic root size to BSA, a ratiometric ($y = a * BSA$) equation was first employed. When a ratiometric equation failed to remove the impact of BSA across all measures, an allometric equation ($y = a * BSA^b$) was subsequently employed (Data Supplement 4). To ensure size independence, fit plots of the residual values over BSA by linear regression determined the presence of residual association.

Preliminary scatter plots, along with lines of best fit and their associated variance (R^2), indicated that chronological age explained the greatest variance (R^2) among the measurements of Doppler and TDI. As per previously published Doppler and TDI velocities Z-score equations in paediatric non-athletes [2], second-order polynomial $(y = a * chronological\ age^2 + b * chronological\ age + c)$ and third-order polynomial $(y = a * chronological\ age^3 + b * chronological\ age^2 + c * chronological\ age\ + \ d)$ equations were employed. To ensure chronological age independence, fit plots of the residual values over chronological age by linear regression determined the presence of residual associations. Conversely, LVEF demonstrated no significant association with BSA or chronological and biological age with a normal distribution, allowing for the determination of a lower refence limit for the calculation of Z-scores [3].

Visual inspection of biological plausibility and the additional amount of variance (R^2) explained by third-order polynomial equations (maximal increase of 1% considered clinically insignificant) contributed to the adoption of a second-order polynomial equation across all Doppler and TDI measures.

When the equations were determined, the residual association between race, chronological and biological age to LV, LA and aortic root size, in addition to HR for Doppler and TDI, were determined by multivariable linear regression. If the interaction with chronological and/or biological age was significant, the allometric equation $y = a * BSA^{(b+c*age)}$ was employed for measures of LV, LA, and aortic root size. If the interaction of race was significant across all measurements, additional variance (R^2) explained by the determination of race-specific constants and coefficients were assessed. As per Lopez et al. [4], an increase of <5% was defined as clinically insignificant.

Preliminary analysis revealed nonconstant variance (heteroscedasticity) of residual values across the entire range of BSA and chronological age, respectively, for most measures of cardiac size, Doppler, and TDI. Accordingly, the regressed SD (RSD) was calculated via the linear regression of the scaled absolute value (multiplied by $\sqrt{(2/\pi)}$) [5]. Z-scores were then calculated with measurements plotted against BSA and chronological age, respectively, with lines depicting the mean and 1Z and 2Z above and below the mean.

References

1. Cavarretta, E.; Maffessanti, F.; Sperandii, F.; Guerra, E.; Quaranta, F.; Nigro, A.; Minati, M.; Rebecchi, M.; Fossati, C.; Calò, L.; et al. Reference Values of Left Heart Echocardiographic Dimensions and Mass in Male Peri-Pubertal Athletes. *European Journal of Preventive Cardiology* **2018**, *25*, 1204–1215, doi:10.1177/2047487318776084.

2. Dallaire, F.; Slorach, C.; Hui, W.; Sarkola, T.; Friedberg, M.K.; Bradley, T.J.; Jaeggi, E.; Dragulescu, a.; Har, R.L.H.; Cherney, D.Z.I.; et al. Reference Values for Pulse Wave Doppler and Tissue Doppler Imaging in Pediatric Echocardiography. *Circulation: Cardiovascular Imaging* **2015**, *8*, e002167, doi:10.1161/CIRCIMAGING.114.002167.

3. Fisher, R. *Statistical Methods for Research Workers*; Fourth.; UK: Oliver & Boyd, 1932;

4. Lopez, L.; Colan, S.; Stylianou, M.; Granger, S.; Trachtenberg, F.; Frommelt, P.; Pearson, G.; Camarda, J.; Cnota, J.; Cohen, M.; et al. Relationship of Echocardiographic Z Scores Adjusted for Body Surface Area to Age, Sex, Race, and Ethnicity: The Pediatric Heart Network Normal Echocardiogram Database. *Circulation. Cardiovascular imaging* **2017**, *10*, 1–7, doi:10.1161/CIRCIMAGING.117.006979.

5. DeVore, G.R. Computing the *Z* Score and Centiles for Cross-Sectional Analysis: A Practical Approach. *Journal of Ultrasound in Medicine* **2017**, *36*, 459–473, doi:10.7863/ultra.16.03025.
